# Magnetic targeting of lornoxicam/SPION bilosomes loaded in a thermosensitive in situ hydrogel system for the management of osteoarthritis: Optimization, in vitro, ex vivo, and in vivo studies in rat model via modulation of RANKL/OPG

**DOI:** 10.1007/s13346-023-01503-8

**Published:** 2023-12-29

**Authors:** Basma Ibrahiem, Rehab Shamma, Abeer Salama, Hanan Refai

**Affiliations:** 1 Department of Pharmaceutics, College of Pharmaceutical Sciences and Drug Manufacturing, Misr University for Science and Technology, Giza, 12566 Egypt; 2 Department of Pharmaceutics and Industrial Pharmacy, Faculty of Pharmacy, Cairo University, El-Kasr El-Aini Street, Cairo, 11562 Egypt; 3 Department of Pharmacology, National Research Centre (NRC), Giza, 12622 Egypt

**Keywords:** Osteoarthritis, Lornoxicam, Superparamagnetic iron oxide nanoparticles, Magnetic joint targeting, Bilosomes

## Abstract

Osteoarthritis is a bone and joint condition characterized pathologically by articular cartilage degenerative damage and can develop into a devastating and permanently disabling disorder. This investigation aimed to formulate the anti-inflammatory drug lornoxicam (LOR) into bile salt–enriched vesicles loaded in an in situ forming hydrogel as a potential local treatment of osteoarthritis. This was achieved by formulating LOR-loaded bilosomes that are also loaded with superparamagnetic iron oxide nanoparticles (SPIONs) for intra-muscular (IM) administration to improve joint targeting and localization by applying an external magnet to the joint. A 3^1^.2^2^ full factorial design was employed to develop the bilosomal dispersions and the optimized formula including SPION (LSB) was loaded into a thermosensitive hydrogel. Moreover, in vivo evaluation revealed that the IM administration of LSB combined with the application of an external magnet to the joint reversed carrageen-induced suppression in motor activity and osteoprotegerin by significantly reducing the elevations in mitogen-activated protein kinases, extracellular signal-regulated kinase, and receptor activator of nuclear factor kappa beta/osteoprotegerin expressions. In addition, the histopathological evaluation of knee joint tissues showed a remarkable improvement in the injured joint tissues. The results proved that the developed LSB could be a promising IM drug delivery system for osteoarthritis management.

**Graphical Abstract:**

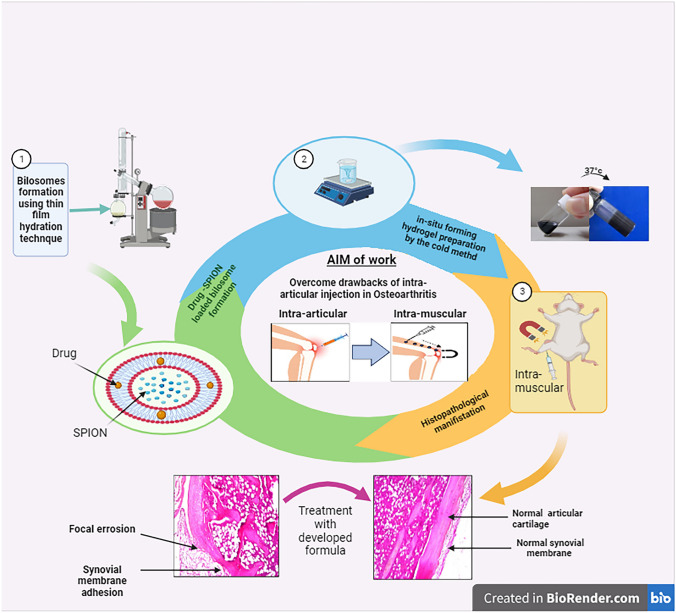

## Introduction

Osteoarthritis (OA), also denominated as degenerative arthritis or hypertrophic arthritis, is a common, inflammatory, degenerative, and debilitant joint disorder. It mostly affects moveable joints, such as the hip and knee, developing structural alterations such as osteophyte creation, subchondral bone remodeling, synovial inflammation, and progressive articular cartilage deterioration [1, 2]. Cartilage is devoid of nutritional paths such as blood vessels and nerves, and it is constructed from only one cell type with minimal proliferative capacity so it is extremely difficult to restore itself once destroyed [3]. The most common complaints are chronic pain, crepitus, stiffness-restricted mobility caused by the eruption, and loss of cartilage elasticity that secures easy movement of the knee joint. These symptoms severely impair the patient’s quality of life and physical function [4, 5]. In order to assess the severity of the illness as well as to evaluate the effectiveness and safety of medications that treat osteoarthritis, different biomarkers that submit useful diagnostic and prognostic techniques can be used. Such biomarkers include cytokines like interleukin (IL)-6, IL-15, and IL-1β, tumor necrosis factor-α (TNF-α), NF-κβ, and vascular endothelial growth factor [6] in addition to mitogen-activated protein kinases (MAPKs) and extracellular signal-regulated kinase (ERK). Furthermore, it is reported that receptor activator of nuclear factor kappa beta (RANKL) plays an important role in bone resorption, while osteoprotegerin (OPG) inhibits the reduction of this bone resorption [7].

Non-steroidal anti-inflammatory drugs (NSAIDs) are the first-choice therapy option for controlling both inflammation and symptomatic pain since inflammatory mediators strongly influence the progress of OA [8–10]. Lornoxicam (LOR) belongs to the oxicam family of NSAIDs and is commonly indicated in the management of osteoarthritis, owing to its extremely potent anti-inflammatory action, pain relief effect, prevention of bone devastation in polyarthritic disorder, and promotion of cartilage formation [11–13]. LOR is classified as BCS category II, as a result of its poor aqueous solubility (18 mg/L) along with high permeability [14]. Its maximum plasma concentration is accomplished within 2.5 h, while the short half-life fluctuates between 3 and 5 h necessitating frequent dosing [15].

For the management of osteoarthritis, the anti-inflammatory drug is usually administered by intra-articular injection. Intra-articular administration has the potential to offer localized drug delivery to afflicted tissues, consequently enhancing local drug bioavailability, and minimizing joint pain and inflammation [9, 16, 17]. However, this route is limited by rapid decay and clearance of injected therapeutic agents resulting in the increased need for frequent injection and also increases the risk of complications such as joint infection [2, 18], besides being a painful injection that needs a professional for its administration [16, 19]. Therefore, avoiding the intra-articular administration and at the same time targeting the dug to the joints seem to be a very interesting approach to manage OA.

Nanocarriers are recently used to deliver the drug efficiently to target site by various techniques. Among the promising nanocarriers are bilosomes. Bilosomes are colloidal bilayer structures comprised of lipid similar to liposomes with integrated bile salts [20, 21]. In drug delivery, bile salts are natural permeability enhancers by increasing the solubility of lipophilic molecules and the fluidity of biological barriers, which allows bilosomes to pass through biological membranes and furnish the bilosomes with an extremely flexible and deformable character in contrast to liposomes [22, 23]. Such permeability enhancement results in the maximization of drug bioavailability. Remarkably, bilosomes have been capable of overcoming multiple challenges experienced by traditional liposomes as leakage of capsulated agent on storage and limited stability [24]. Bilosomes function not only as drug transporters but also as drug localizers resulting in increasing the local concentration and activity of the therapeutic agent [25].

In order to reduce the frequency of drug administration, the nanocarrier dispersion is preferably administered in a dosage form of higher viscosity to slow the rate of transportation of the nanoparticles. In situ forming hydrogels combine the profits of a solution in terms of dosage precision and convenience of administration with those of a gel in terms of extending the drug retention period [26]. One of the most widely used stimuli-sensitive hydrogels is a thermosensitive hydrogel, which may be made by combining the hydrogel with medications and does not need the addition of crosslinking agents, organic solvents, or complicated techniques like chemical synthesis [27]. Magnetic targeting is one of the interesting techniques to target the nanocarriers to site of action. Superparamagnetic iron oxide nanoparticles (SPIONs) can enhance the targeting of nanocarrier loaded with the drug by guiding it to the site of action with the aid of an external magnetic field [28]. SPIONs provide a number of benefits, including ease of fabrication, biocompatibility, possibility of surface functionalization, and the capacity for accurate remote control without leaving a residual magnetic impact after the elimination of the external magnetic field [29]. Drug-SPION coupling for magnetic drug delivery can be accomplished either by binding the drug directly to the iron oxide surface or by encasing drugs as well as SPIONs within nanocarriers [30].

The present study aimed to target and localize LOR delivery in the joints after intra-muscular administration in the thigh muscle instead of the intra-articular administration of the drug for enhancing the patient’s compliance, overcoming the drawbacks of intra-articular administration, and facilitating the administration without the need for a specialist. To achieve this, target bilosomes loaded with LOR and SPIONs were prepared and characterized. The optimized formula was incorporated into a thermosensitive in situ forming hydrogel and was tested in vivo by injecting the formula into the thigh muscle of rats with carrageenan-induced osteoarthritis combined with the application of an external magnet directed to the knee to direct the particles to the joint. The anti-arthritic effect of the formula was evaluated in comparison to the free unencapsulated drug by several techniques including testing the modulation of RANKL/OPG pathway. According to our knowledge, this pathway was not tested before for LOR.

## Materials and methods

### Materials

Lornoxicam (LOR) was kindly gifted by Delta Pharma, 10th of Ramadan City, Egypt. Sodium taurocholate hydrate (STC) was purchased from Sigma-Aldrich, Taufkirchen, Germany. Sodium deoxycholate (SDC) and sodium cholate hydrate (SC) were purchased from Alfa Aesar, Karlsruhe, Germany. Sorbitan monostearate (Span^®^ 60) was provided from Merck Schuchardt OHG, Hohenbrunn, Germany. Hyaluronic acid (HA) was supplied by DSM personal care and Beauty (Basel, Switzerland). Synperonic™ PE/F 127 (PE/F127), cholesterol (CHL), carrageenan, polyoxyethylene sorbitan monostearate (Tween^®^ 60), and cellulose dialysis membrane (12,000–14,000 molecular weight cutoff) were purchased from Sigma-Aldrich Corp., St. Louis, MO, USA. Other used chemicals were as follows: chloroform, HPLC grade (Fisher Scientific, UK), ammonium hydroxide (El Salam for Chemical Industries, Egypt), and methanol (Lopa, Mumbai, India), potassium dihydrogen phosphate, dipotassium hydrogen phosphate, and sodium chloride (El-Gomhoria for Chemistry Industrial, Giza, Egypt). Ferrous sulfate tetrahydrate and ferric chloride hexahydrate were purchased from Alpha Chemika.com, Mumbai, India. Mitogen-activated protein kinase (MAPK), extracellular signal-regulated kinase (ERK1), receptor activator of nuclear factor kappa beta (RANKL), and osteoprotegerin (OPG) were assessed by Sunlong Biotech Co., Ltd, China, ELISA (enzyme-linked immunosorbent assay) kit.

### Methods

#### Preparation of LOR-loaded bilosomes

The fabrication of LOR-loaded bilosomes was accomplished using the thin film hydration approach by altering both the type and amount of bile salt and the type of surfactant [31]. Briefly, LOR (16 mg), 125 mg of the surfactant (Tween 60 or Span 60), and 25 mg of CHL were precisely weighed and dissolved in 10 mL of chloroform with various amounts of the employed bile salt (SC, SDC, or STC) utilizing an ultrasonic bath sonicator for 10 min [32]. The resultant clear organic solution was then transferred to a 250-mL round bottom flask and slowly evaporated at 60 °C under reduced pressure using a rotary evaporator (rotatory evaporator, Model Heidolph rotavapor vv 2000/WB 2000, Germany) for 30 min at 120 rpm until a thin, fully dry film was created [33].

Using the same equipment and normal pressure, the dry film was then hydrated with 10 mL PBS (pH = 7.4) by spinning the flask in a water bath maintained at 60 °C for 30 min at 150 rpm. Glass beads were utilized in the hydration stage to maximize the yield of the created nanovesicles [34]. The resulting large vesicle dispersion was then smashed up into smaller ones by sonication for 3 min in a bath sonicator (Ultra-Sonicator, Model LC 60/H; Elma, Germany) at 25 °C [35]. Finally, to ensure full annealing of vesicles and partitioning of the drug between the aqueous core and bilayer, the resulting fine-tuned dispersion was allowed to equilibrate overnight at 4 °C [36].

#### Evaluation and optimization of the prepared LOR-loaded bilosomes

##### Determination of LOR entrapment efficiency percent

The percentage of entrapped LOR was calculated indirectly by measuring the unentrapped LOR in the dispersion medium. One milliliter of the dispersion medium was centrifuged via a cooling centrifuge (refrigerated centrifuge, Model 3 K 30, Sigma, Germany) at 15,000 rpm for 1 h at 4 °C [33]. The supernatant was withdrawn, and the residue was then washed with 10 mL PBS and re-centrifuged. The supernatant was separated, and then, the unentrapped drug content in the supernatant was examined spectrophotometrically at *λ*max 376 nm [37] using Shimadzu UV spectrophotometer (Model UV - 1650 P.C., Japan). Each result was the mean of three determinations ± standard deviation (SD).

The entrapment efficiency (EE%) was calculated by subtracting the free LOR in the supernatant from the total drug incorporated using the following equation:

$$\mathrm{EE}\%=\frac{\mathrm T\mathrm o\mathrm t\mathrm a\mathrm l\;\mathrm a\mathrm m\mathrm o\mathrm u\mathrm n\mathrm t\;\mathrm o\mathrm f\;\mathrm L\mathrm O\mathrm R-\mathrm U\mathrm n\mathrm e\mathrm n\mathrm t\mathrm r\mathrm a\mathrm p\mathrm p\mathrm e\mathrm d\;\mathrm L\mathrm O\mathrm R}{\mathrm T\mathrm o\mathrm t\mathrm a\mathrm l\;\mathrm a\mathrm m\mathrm o\mathrm u\mathrm n\mathrm t\;\mathrm o\mathrm f\;\mathrm L\mathrm O\mathrm R}\times100$$

##### Determination of particle size and zeta potential of LOR-loaded bilosomes

The average particle size and zeta potential of the prepared LOR-loaded bilosomes were estimated by dynamic light scattering process at 25 ± 2 °C employing a helium–neon laser using a Zetasizer (Malvern Instrument Ltd., Worcestershire, UK). Before every measurement, the bilosomal dispersions were adequately diluted by deionized water to ensure that the light scattering amplitude was within the instrument’s sensitivity range. The same equipment was used to measure zeta potential to detect the particles’ electrophoretic motion in an electric field. Analysis time was maintained at 70 s and three replicates were taken for every sample. The data are displayed as the average value ± SD.

##### Studying the effect of different formulation variables using 3^1^.2^2^ full factorial design

Using Design-Expert^®^ software version 13 (Sat-Ease, Inc., Minneapolis, MN), a 31.22 full factorial design was exploited to assess the impact of various parameters in formulating LOR-loaded bilosomes. In this design, three independent variables were analyzed: X1: the type of surfactant with two levels, X2: the type of bile salt with three levels, and X3: the quantity of bile salt with two levels. Their influence on entrapment efficiency (%, *Y*1), particle size (nm, *Y*2), and zeta potential (mV, *Y*3) as a dependent parameter was observed (Table 1). All conceivable combinations for preparing LOR-loaded bilosomes are displayed in Table 2. The design illustrates the influence of the independent factors individually (*X*1 or *X*2 or *X*3) and their interaction effect (*X*1*X*2,* X*1*X*3, *X*2*X*3) on the particle size, entrapment efficiency percent, and zeta potential.

**Table 1:** The levels of independent variables and the model summary statistics of 3^1^.2^2^ full factorial design used for the optimization of LOR-loaded BLs

**Factors (independent variables)**	**Levels of variables**							
***X***_**1**_**: Surf type**				S60				T60
***X***_**2**_**: BS type**				SDC		SC		STC
***X***_**3**_**: BS amount (mg)**				5				15

**Responses (dependent variables)**	***R***^**2**^	**Adjusted *R***^**2**^	**Predicted *R***^**2**^	**Constraints**	***p*-value**	***F* value**	**Adequate precision**	**Significant factors**
***Y***_**1**_**: EE%**	0.9893	0.9852	0.9753	Maximize	< 0.0001	286.59	49.6	*x*_1_, *x*_2_, *x*_3_
***Y***_**2**_**: PS (nm)**	0.9711	0.9611	0.9445	Minimize	< 0.0001	96.97	31.3	*x*_1_, *x*_2_, *x*_3_
***Y***_**3**_**: ZP (mV)**	0.9739	0.9649	0.9500	Maximize (absolute value)	< 0.0001	107.96	28.68	*x*_1_, *x*_2_, *x*_3_

*BS* bile salt, *Surf* surfactant, *EE%* entrapment efficiency percent, *BLs* bilosomes, *PS* particle size, *SDC* sodium deoxycholate, *SC* sodium cholate, *STC* sodium taurocholate, *ZP* zeta potential

**Table 2:** (A) Experimental runs, independent variables, and measured responses of the 3^1^.2^2^ full factorial experimental design of LBs and (B) the observed, predicted values, and bias percent of the optimum LB4

**Formulations**	***X***_**1**_	***X***_**2**_	***X***_**3**_	**Y1 (EE%)**	**Y2 PS (nm)**	**Y4 ZP (mV)**
**LB1**	S60	SC	5	85.79 ± 1.79	312 ± 4	− 41.63 ± 0.70
**LB2**	S60	SC	15	85.55 ± 0.76	277 ± 10	− 40.73 ± 0.29
**LB3**	S60	SDC	5	90.01 ± 0.67	381 ± 11	− 44.33 ± 1.08
**LB4**	S60	SDC	15	87.10 ± 1.67	254 ± 14	− 40.23 ± 0.12
**LB5**	S60	STC	5	84.79 ± 1.60	297 ± 2	− 40.70 ± 0.78
**LB6**	S60	STC	15	71.60 ± 1.05	248 ± 5	− 40.07 ± 0.69
**LB7**	T60	SC	5	56.44 ± 2.26	169 ± 8	− 28.00 ± 0.83
**LB8**	T60	SC	15	54.99 ± 1.26	140 ± 21	− 24.27 ± 1.14
**LB9**	T60	SDC	5	65.55 ± 0.71	286 ± 23	− 29.90 ± 1.14
**LB10**	T60	SDC	15	57.01 ± 0.83	173 ± 14	− 24.63 ± 1.77
**LB11**	T60	STC	5	53.10 ± 3.07	146 ± 10	− 22.80 ± 3.61
**LB12**	T60	STC	15	47.95 ± 2.25	120 ± 6.28	− 17.77 ± 1.28
**LSB**	S60	SDC	15	87.24 ± 0.85	323 ± 7.20	− 32.50 ± 1.60
**Observed values of LB4**				87.1	254	− 40.2
**Predicted values of LB4**				86.2	254	− 40.6

Data are presented as mean ± SD (*n*= 3)

*X*_*1 *_surf type, *X*_*2*_ BS type, *X*_*3*_ BS amount, *Y*_*1*_* (EE%)* entrapment efficiency percent (%), *Y*_*2*_*(PS)* particle size (nm), *Y*_*3*_*(ZP)* zeta potential (mV), *LBs*LOR-loaded bilosomes

##### Optimization of LOR-loaded bilosomes

The desirability function in Design-Expert^®^ software was used to choose the optimum bilosomes. The goal of the optimization procedure was to select a system for LOR-loaded bilosomes with maximum entrapment efficiency percent as well as minimum particle size. The response with a desirability factor near 1 was adopted. The recommended LOR-loaded bilosomes were developed, evaluated, and compared to the anticipated responses to validate the model [37, 38].

#### Preparation of superparamagnetic iron oxide nanoparticle

The SPION was synthesized using the coprecipitation technique, as previously described by Abbas et al. [30]. In brief, ferrous sulfate tetrahydrate (0.6 g) and ferric chloride hexahydrate (1.17 g) were dissolved independently in 50 mL of deionized water within a nitrogen atmosphere in a molar ratio of 1:1.75, respectively, by vigorous agitation. Both solutions were mixed together at 70 °C for 1 h. The mixture was infused with ammonium hydroxide (32%), stirred for an additional hour, and then chilled to room temperature. The solution’s color transformed from yellow to black, suggesting the development of magnetite nanoparticles. Lastly, the generated nanoparticles were pulled out of the solution using a magnet, rinsed five times with hot water, and dried overnight in a 50 °C oven (Natural Convection Oven LDO-080N, Korea).

#### Preparation of LOR/SPION-loaded bilosomes

LOR/SPION-loaded bilosomes (LSB) were prepared as described above in the preparation of LOR-loaded bilosomes, with a slight modification. The dry film of the selected formula was hydrated with an adequate quantity of PBS/aqueous ferrofluid, namely700 μL of 10.3 mg/mL of SPION [29] mixture to produce a final volume of 10 mL [39, 40].

#### Characterization of LSB

##### Evaluation of entrapment efficiency percent, particle size, and zeta potential

The entrapment efficiency percent of LOR in the prepared LSB, the particle size, and zeta potential were evaluated using the same methods mentioned earlier for the evaluation of LOR-loaded bilosomes.

##### Evaluation of the magnetism

At ambient temperature, LSB’s magnetization was measured with a vibrating sample magnetometer (VSM) (Lake Shore Model 7410, USA).

#### Morphological screening of the optimized LOR-loaded bilosome (LB4) and LSB

The optimized LOR-loaded bilosome (LB4) and LSB were morphologically investigated using a transmission electron microscope (TEM) (Joel JEM 1230, Tokyo, Japan). A drop of the dispersion was applied to a carbon-coated copper grid that has been mounted on filter paper to soak up any surplus, and the grid was then left to dry into a thin film. Before this film on the grid dried completely, one drop of the freshly prepared stain was applied and allowed to air dry. After dryness, the samples were then inspected using the TEM. Photographs were captured with the appropriate magnification [41].

#### Lyophilization of the optimized LOR-loaded bilosomes (LB4) and LSB

Before the lyophilization process, the optimized LOR-loaded bilosome (LB4) and LSB were refrigerated at − 18 °C for 24 h and then lyophilized by freezing it at a specified temperature (− 45 °C) and pressure of 0.07 mbar for 24 h to transform it into a powder (Labconco™ Cascade Free Zone 6 Plus™ Freeze-Dry Systems, US Models, USA) [33].

#### Differential scanning calorimetry (DSC)

DSC calibrated with indium was used to investigate the thermal behavior of the optimized LOR-loaded bilosome (LB4) and LSB. Thermal characterization was accomplished on optimized lyophilized LOR-loaded bilosomes (LB4) and LSB along with individual components: LOR plain powder, Span^®^60, cholesterol, and SDC using DSC (Shimadzu Corporation, DSC-60 with the thermal analyzer, TA-60 WS thermal analyzer, Shimadzu, Tokyo, Japan). A specified amount of each sample was heated over a temperature range of 20 to 300 °C at a scanning rate of 5 °C/min with inert nitrogen inflow (25 mL/min) [32].

#### Stability study

To assess the impact of storage on the optimized LOR-loaded bilosome (LB4) and LSB, a sample was maintained in a tightly sealed glass vial at (4 °C) for 90 days. The entrapment efficiency percent, particle size, and zeta potential of the sample were evaluated at the end of the storage duration relative to the first measurements of freshly prepared formulations. All measurements were carried out in triplicate. SPSS^®^ software 22.0 (SPSS, Chicago, IL, USA) was employed to statistically analyze the findings using the paired *t*-test [25].

#### Preparation of LSB in situ forming hydrogels

LSB in situ forming hydrogels were prepared by the cold method using Synperonic™ PE/F 127 (PE/F 127) as a thermosensitive polymer, in addition to hyaluronic acid (HA). Precisely weighed quantities of PE/F 127 and HA were dispersed under constant stirring in the cold bilosomal dispersion, equilibrated at 4–6 °C using an ice bath, using a magnetic stirrer (model MSH-20D, GmbH, Germany) for 2 h. The dispersion was kept in a refrigerator (4 °C) for at least 24 h to ensure the complete removal of air bubbles and full dissolution of the components [42].

#### Evaluation of the developed in situ forming hydrogel

##### Assessment of the gelation temperature

The test tube inversion method was used to determine the gelation temperature of the prepared in situ forming hydrogels [43]. Briefly, glass vials each containing 2 mL of the investigated systems were submerged in a thermostatically monitored water bath. The temperature was elevated gradually at a rate of 0.5 °C/min from 20 to 40 °C, and at each set point, the test tube was turned upside-down at 90°. The gelation temperature was defined as the temperature at which no flow was observed upon inversion of the vial.

##### Assessment of gelation time

The gelation time of the developed LSB in situ forming hydrogels was also evaluated using the test tube inversion method [42]. A test tube containing 2 mL of the chosen LSB in situ forming hydrogel was submerged in a thermostatic water bath maintained at 37 ± 0.5 °C. The gelation time is the time required to transform the formula from a liquid state into a gel with no evidence of flow upon inversion of the test tube.

##### Assessment of viscosity and rheological property

The viscosity of the optimized LSB in situ forming hydrogel (LSB4c) was investigated utilizing a cone and plate viscometer (Brookfield viscometer; type DVT2, Brookfield Engineering Labs., Middleborough, MA). Exactly, 0.5 mL of the optimized LSB in situ forming hydrogel (LSB4c) was dropped into the cup plate, and the space between the cone and plate was adjusted. To investigate the impact of temperature on the viscosity of in situ forming hydrogel, the CP-52 (Cone/Spindle 52) revolved at a steady speed of 10 rpm, and the apparent viscosity was measured at two different temperatures (4 °C and 37 °C). The rheological property was investigated by allowing the dispersion to convert into a gel at 37 °C. Afterward, the determination of viscosity was conducted at various angular velocities (10, 20, 30, 40, and 50 rpm) with 10 s elapsed between each pair of subsequent speeds, and it was then repeated in descending order of velocities. A rheogram (graph between shear rate and the corresponding viscosities) was plotted to depict the formulation’s flow pattern [44].

##### Syringeability study

The capability of the developed formulation to flux easily through a 21-G needle-equipped syringe was evaluated by filling the syringe with 1 mL of the cold optimized LSB in situ forming hydrogel (LSB4c) and gentle pressure was applied on the syringe’s injector [45, 46].

#### In vitro release study

The dissolution profile of LOR was ascertained from LOR suspension, the optimized LOR-loaded bilosome (LB4), LSB, and the optimized LSB in situ forming hydrogel (LSB4c) through the employment of a shaking water bath (shaking water bath, model LSB-O15S Labtech). Briefly, a specified quantity of each formula was loaded in a previously treated dialysis bag by soaking it in dissolution media overnight (dialysis tubing cellulose membrane, Sigma-Aldrich Co., St. Louis, USA; molecular weight cutoff 12,000–14,000). The bags were sealed on both ends to prevent leakage and then hanged in screw-capped bottles, which were packed with 95 mL of phosphate buffer saline (PBS) with pH 7.4 to retain a sink condition. The experiment was implemented at 50 rpm, and at 37 ± 0.5 °C in a shaking water bath. To preserve the sink condition, 4-mL samples were drawn at fixed time intervals (0.5, 1, 2, 4, 6, 8, 10, 12, 24, and 48 h) and immediately replenished with the same volume of fresh dissolution media. The LOR content of the samples was evaluated spectrophotometrically at 376 nm [29, 47]. Using SPSS^®^ software 22.0 (SPSS, Chicago, IL, USA), a statistical analysis of the rate of LOR release from various systems was carried out using univariate and Tukey’s post hoc test. Additionally, in order to evaluate the release mechanism from various systems, the gathered data were fitted into several model equations using the DDSolver software (Excel Add-in). The highest correlation coefficient (*R*^2^) was used to determine which model fitted the data the best [48].

#### In vivo studies

##### Animal housing and handling

Wister albino male rats (140–150 g) were provided by the Animal House of the National Research Centre (Cairo, Egypt). The rats were housed under temperature- and light-controlled conditions (24 ± 2 °C under a 12-h light/dark cycle) and had free access to standard food and water. The animal experiments were performed in accordance with the guidelines of the Institutional Animal Ethics Committee (Medical Research Ethics Committee) of the NRC (National Research Center), Cairo, Egypt, that adhere to ARRIVE guidelines.

##### Experimental design of the in vivo study

The induction of arthritis was achieved by intra-articular injection of carrageenan (0.02 mL/joint) into the rats’ right knees for 10 days [49]. Rats were assigned into five groups, each containing eight male rats as follows: group 1: normal control, group 2: positive control (carrageenan) group, group 3: rats treated with intra-muscular injection (thigh muscle) of the in situ forming hydrogel containing the free drug (4 mg/kg) [50], groups 4 and 5: rats treated with intra-muscular injection of in situ forming hydrogel of the optimized LOR-loaded bilosome (LB4) and the optimized LSB in situ forming hydrogel (LSB4c), respectively. The treatment continued for 10 days, concurrent with carrageenan.

##### Evaluation of the joint diameter

Knee joint thickness was measured under anesthesia using an electronic digital caliper (Mitutoyo, Japan).

##### Evaluation of the locomotor activity and coordination

Motor activity was measured by evaluating rat movements using a grid floor activity cage (model no. 7430, Ugo Basile, Italy). Rats were acclimatized for 1 h to the test room, before placing the animal in the activity cage (exposure) [51]. The activity counts of rats were measured in three successive sessions, each of 5-min duration, before beginning the experiment to habituate the rats to the apparatus [52]. Then, the rats were placed in the activity cage and the activity counts were measured over 5-min durations at the end of the experiment [53].

##### Tissue sample preparation

At the end of the experimental period, rats were anesthetized using pentobarbital sodium (40 mg/kg, IP) and then sacrificed by decapitation. A sample of the knee joint was removed from all rats, and then homogenized to obtain 20% homogenate. The homogenate was centrifuged for 10 min using a cooling centrifuge at 3000 rpm; the supernatant was taken for the estimation of knee joint biochemical parameters.

##### Evaluation of MAPK/ERK1 and RANKL/OPG signaling pathway

Mitogen-activated protein kinase (MAPK), extracellular signal-regulated kinase (ERK1), receptor activator of nuclear factor kappa beta (RANKL), and osteoprotegerin (OPG) were assessed by Sunlong Biotech Co., Ltd, China, ELISA kit. The manufacturer’s instructions of the kit were followed for evaluating the results. Samples and standards were placed into the wells with immobilized antibodies specific for rat MAPK, ERK1, RANKL, and OPG and then were incubated. Biotinylated antirat MAPK, ERK1, RANKL, and OPG antibodies were added after incubation and washing. Any unbound substances were removed by washing, and horseradish peroxidase–conjugated streptavidin was placed into the wells, which were washed once again. TMB (tetramethyl benzidine) substrate solution was added to the wells; color developed proportionally to MAPK, ERK1, RANKL, and OPG bound amount. Color development was discontinued (stop solution) and the color intensity was measured at 450 nm [54].

##### Statistical evaluation

All the values are presented as means ± standard deviation of the means (SD). Comparisons between diverse groups were carried out using one-way analysis of variance (ANOVA) followed by Fisher’s LSD test for multiple comparisons (GraphPad Prism software, version 5 (Inc., USA)). Statistical difference (*p* < 0.05) was considered significant.

##### Histopathological examination

Twenty-one days post carrageenan injection, rats were anesthetized using pentobarbital sodium anesthesia (40 mg/kg, IP) and sacrificed. Autopsy samples were taken from the knee joint of rats in different groups and fixed in 10% formol saline for 24 h and decalcified by 10% formic acid. Washing was done in distilled water; then, serial dilutions of alcohol (methyl, ethyl, and absolute ethyl) were used for dehydration. Specimens were cleared in xylene and embedded in paraffin at 56 °C in a hot air oven for 24 h. Paraffin beeswax tissue blocks were prepared for sectioning at 4-μm thickness by rotary LEITZ microtome. The obtained tissue sections were collected on glass slides, deparaffinized, and stained by hematoxylin and eosin stain (HE) [55] for examination through the light electric microscope.

## Results and discussion

### Statistical analysis of the experimental design

Design of experiments (DOE) is a useful approach for studying the combined impact of formulation variables on vesicles’ properties with the least number of runs in which several independent variables may be altered to study their effect on various responses [56]. The model adopted was two-factor interaction (2 FI).

All the investigated variables had high *R*2 values and significant correlations between the predicted and adjusted *R*2, since each of them exhibited a difference of less than 0.2 (Table 1). The ratio of signal to noise was measured with adequate precision to guarantee that this model can be used to navigate the design space. A ratio greater than four is preferred [57] which was observed for all responses as shown in Table 1.

### Impact of formulation variables on entrapment efficiency percent (*Y*_1_) of LOR in LOR-loaded bilosomes

**Fig. 1:**
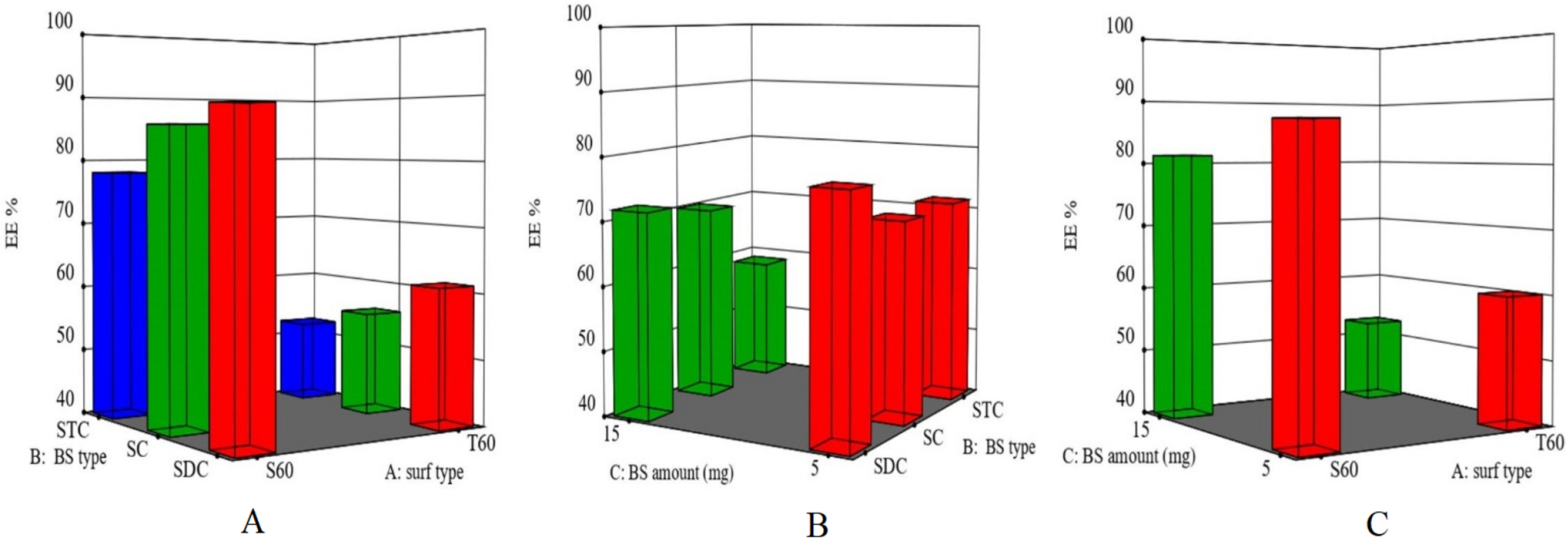
Response 3-D plots for the effect of **A** surf type (*X*_1_), **B** BS type (*X*_2_), and **C** BS amount (*X*_3_) on EE% of LOR-loaded BLs. Abbreviations: BS, bile salt; Surf, surfactant; EE%, entrapment efficiency percent; LOR; lornoxicam; and BLs, bilosomes

Results indicated that all formulations had a good capacity to trap LOR. The average entrapment efficiency percent of LOR in different bilosomes ranged from 47.95 ± 2.25 to 90 ± 0.67% as shown in Table 2. The impacts of surfactant type (*X*1), bile salt type (*X*2), and bile salt quantity (*X*3) on the entrapment efficiency percent of LOR-loaded bilosomes are graphically represented in Fig. 1 as response 3-D plots. The level of significance of each investigated parameter’s impact on the entrapment efficiency percent was assessed using an ANOVA test, which declared that each tested parameter significantly affected the entrapment efficiency percent of LOR in the manufactured vesicles (*p* < 0.0001 for the three variables).

The entrapment efficiency percent of LOR in the prepared LOR-loaded bilosomes was significantly influenced by the type of surfactant involved in their fabrication. LOR-loaded bilosomes containing Span 60 displayed a higher entrapment efficiency percent than those containing Tween 60. This can be ascribed to two reasons. The first reason is the surfactant transition temperature (*T*_*C*_). The phase transition temperature (*T*_*C*_) of Span 60 (solid at room temperature) is greater than that of Tween 60 (viscous liquid or semi-gel at room temperature) resulting in higher entrapment within LOR-loaded bilosomes containing Span 60 [58–60]. Previous studies reported that the higher the *T*_*C*_ of the surfactant, the better its capacity to produce a less permeable bilayer and a more ordered gel structure, which may result in higher drug entrapment efficiency percent values, and vice versa [59, 61, 62]. The second reason is the hydrophilic-lipophilic balance (HLB) value. Span 60 has a lower HLB (HLB = 4.7) than Tween 60 (HLB = 14.9), resulting in more hydrophobicity and a higher ability to entrap a hydrophobic drug like LOR [63].

The type of bile salt (*X*_2_) also has a significant impact on the entrapment efficiency percent of LOR in the prepared LOR-loaded bilosomes (*p* < 0.0001). Among the tested bile salts, LOR-loaded bilosomes containing SDC exhibited the highest entrapment efficiency percent in comparison to those created with other bile salts (SC and STC). This may be attributed to the degree of lipophilicity of the tested bile salts. The order of effective intercalation of the hydrophobic drug, LOR, in the bilayer hydrophobic zone with consequent higher entrapment efficiency percent increased as the hydrophobicity of bile salt increased (i.e., HLB value decline) which serves as a barrier delaying the drug leaking out from vesicles [25, 64]. The results show that SD has the highest lipophilicity (HLB = 16) [65] in comparison to SC and STC (HLB = 18 [66] and 22.1 [25], respectively).

ANOVA results also revealed that increasing the bile salt quantity (*X*_2_) (from 5 to 15 mg) significantly decreased LOR entrapment efficiency percent within the prepared LOR-loaded bilosomes. An increment in the bile salt content would result in the development of mixed micelles within the dispersion medium, which enhances drug miscibility in the dispersion medium and minimizes its entrapment within the bilosomes [67]. Furthermore, elevated concentrations of bile salts have a fluidizing impact on the vesicle’s lipid bilayers, resulting in the loss of the trapped drug [22].

### Impact of formulation variables on particle size (*Y*_2_) of LOR-loaded bilosomes

**Fig. 2:**
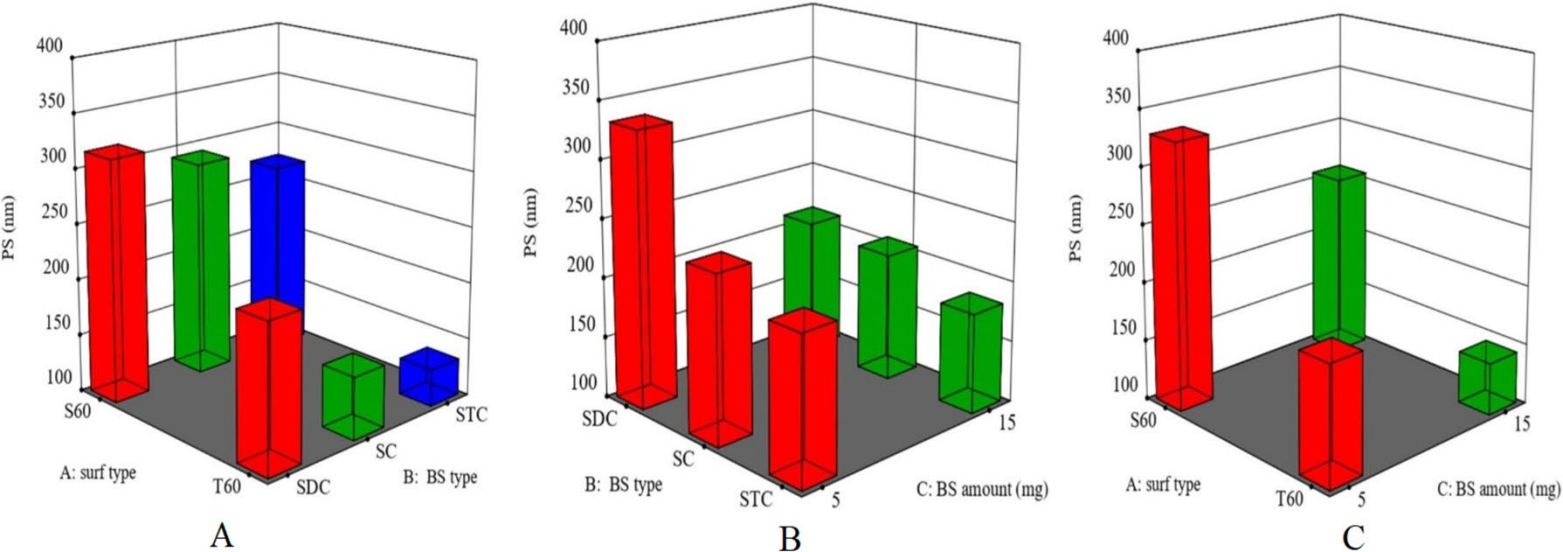
Response 3-D plots for the effect of **A** surf type (*X*_1_), **B** BS type (*X*_2_), and **C** BS amount (*X*_3_) on PS of LOR-loaded BLs. Abbreviations: BS, bile salt; Surf, surfactant; PS, particle size; LOR, lornoxicam; and BLs, bilosomes

All the prepared vesicles were in the nanoscale range, with their particle size ranging from 120 ± 6 to 381 ± 11 nm (Table 2). Response 3-D graphs in Fig. 2 depict the impact of the surfactant type (*X*1), type of bile salt (*X*2), and quantity of bile salt (*X*3) on the particle size of LOR-loaded bilosomes. All tested factors significantly affect the particle size of the manufactured vesicles (*p* < 0.0001 for the three variables). The particle size findings are in agreement with the entrapment efficiency percent.

Concerning the surfactant type, results showed that Span 60–containing LOR-loaded bilosomes showed higher particle size compared to those containing Tween 60. This result correlates well with the results of entrapment efficiency percent, where Span 60–containing LOR-loaded bilosomes showed a higher entrapment efficiency percent compared to those containing Tween 60. Moreover, the HLB value of the surfactant plays an important role in the particle size of the prepared LOR-loaded bilosomes, where an inverse relation between the particle size and HLB is expected. LOR-loaded bilosomes containing a surfactant with a higher HLB value will have higher surface energy and thus reduced particle size. Tween 60 has a higher HLB value (14.9) than Span 60 (4.7). Therefore, the particle size of Tween 60–containing bilosomes is expected to be smaller than that of Span 60–containing ones [68].

Concerning the type of bile salt, results revealed that LOR-loaded bilosomes prepared using STC had the smallest particle size followed by SC and finally SDC. This could be attributed to the amount of drug entrapped within the bilosomes, where the entrapment efficiency percent increases in the same manner.

Regarding the amount of bile salt, results revealed a significant reduction in particle size upon increasing the amount of bile salt (from 5 to 15 mg). This could be attributed to the fact that bile salts are anionic surfactants, which reduce surface tension and raise vesicle flexibility, leading to the creation of smaller vesicles [69]. Another suppositional reason is that mixed micelles, which have a lower particle size than vesicles, develop when the bile salt concentration rises [70].

### Impact of formulation variables on zeta potential (*Y*_3_) of LOR-loaded bilosomes

**Fig. 3:**
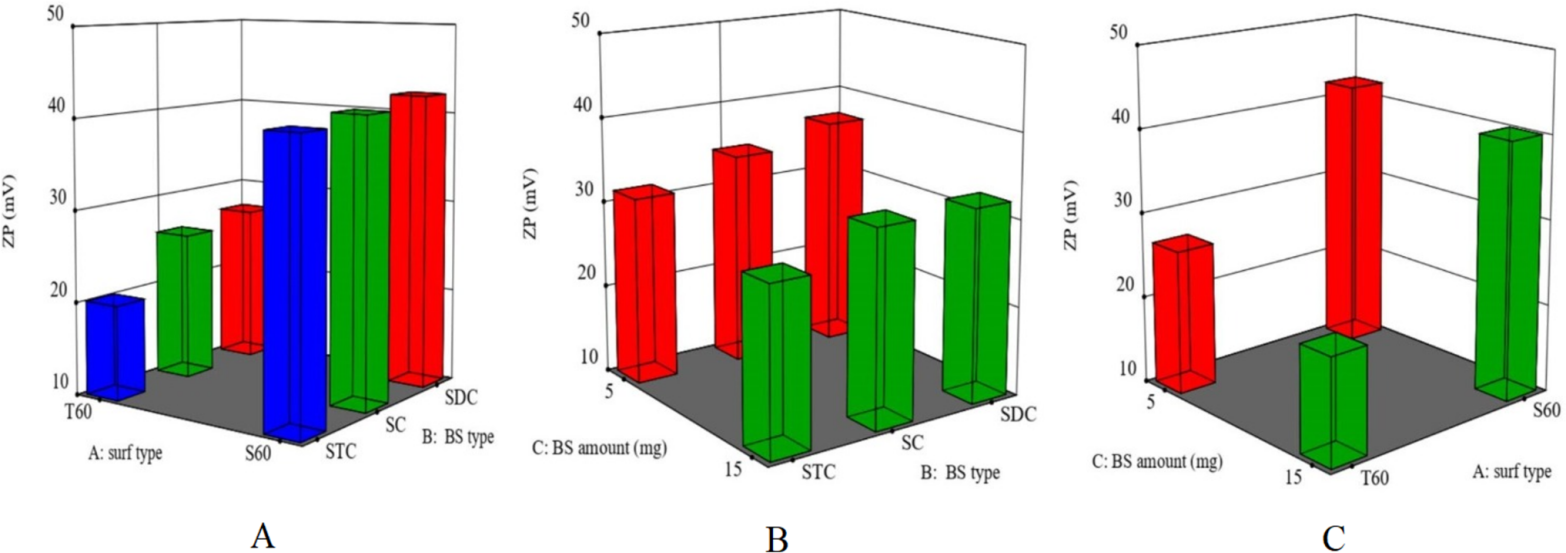
Response 3-D plots for the effect of **A** surf type (*X*_1_), **B** BS type (X_2_), and **C** BS amount (*X*_3_) on ZP of LOR-loaded BLs. Abbreviations: BS, bile salt; Surf, surfactant; ZP, zeta potential; and BLs, bilosomes

The system is stabilized against agglomeration by the magnitude of zeta potential, which reflects the strength of electrostatic repulsion between particles with identical charges in dispersion [71]. All the prepared LOR-loaded bilosomes obtained negative zeta potential values which ranged from −17.8 ± 1.2 to −44.3 ± 1.08 mV (Table 2). These results confirm that LOR-loaded bilosomes have enough charges to prevent them from aggregating and fusing. Since the zeta potential values for all the prepared LOR-loaded bilosomes were negative, the absolute values (without the negative sign) will be utilized in the discussion to avoid misunderstanding. The influence of formulation variables is demonstrated as response 3-D plots in Fig. 3. In accordance with the studied design, zeta potential is significantly affected by the analyzed factors *X*_1_, *X*_2_, and *X*_3_ (*p* < 0.001).

The type of surfactant (*X*_1_) significantly impacted the zeta potential of the prepared bilosomes. Results revealed that Span 60–containing bilosomes demonstrated greater zeta potential values, which may be attributed to its higher LOR entrapment efficiency values compared to those of Tween 60–containing ones. LOR is an acidic drug that ionizes and acquires a negative charge at neutral or alkaline pH. Consequently, its high concentration in the vesicles contributes to the rise in the charge density of bilosomes [72].

The type of bile salt (*X*_2_) also had a significant impact on the zeta potential of the prepared LOR-loaded bilosomes and correlates well with the results of entrapment efficiency percent. LOR-loaded bilosomes containing SDC as a bile salt showed a greater vesicular bilayer charge when compared to SC and STC. This might be clarified by the higher lipophobic feature of STC (HLB = 22.1) followed by SC (HLB = 18) as compared with SDC (HLB = 16) which can result in additional insulating the negative charge by the residence on the vesicular bilayer surface contributing to the disguising of its charge. As a result, the zeta potential value has drastically decreased [67]. The zeta potential of SC-containing bilosomes is lower than that of SDC-containing bilosomes which may be explained by the extra hydroxyl group in SC’s structure compared to SDC’s structure. The observed zeta potential might be impacted by the steric hindrance that could arise from this hydroxyl group [73]. STC gives the least value of zeta potential because of the significant acidity of the taurine group. High STC ionization will discharge more sodium ions into the solution, increasing the electrolyte concentration and, as a result, compressing the bilayer because of the accumulation of counterions which, in turn, mitigates the surface charges, resulting in lower zeta potential values compared to SC and SDC [63, 74].

Concerning the amount of bile salt (*X*_3_), the findings showed a significant reduction in the values of zeta potential upon increasing the bile salt amount. This might be illustrated by the fact that exceeding a particular threshold for bile salt causes the electrostatic double layer to collapse, leading to a considerable decline in zeta potential [38].

### LOR-loaded bilosome optimization

To figure out the level of each independent variable needed for optimization, the optimization process has been carried out for *X*_1_ (type of surfactant), *X*_2_ (type of bile salt), and *X*_3_ (concentration of bile salt) using the following target ranges: maximum entrapment efficiency percent (*Y*_1_), minimum particle size (*Y*_2_), and maximum zeta potential as an absolute value (*Y*_3_).

The optimal values of the variables were obtained graphically and numerically utilizing the Design-Expert software. The system obeying these criteria was the LOR-loaded bilosomes, containing Span 60 and SDC (15 mg), with a desirability value of 0.722. Consequently, this optimized system (LB4) has been selected for additional studies.

### Characterization of LSB

#### Particle size, zeta potential, and entrapment efficiency percent

The synthesized SPIONs had a small particle size (25.0 ± 5.0 nm) and a positive surface charge with a zeta potential of 24.8 ± 0.96 mV. Loading the optimized LOR-loaded bilosome (LB4) with SPION resulted in a significant increase in the particle size and a decrease in zeta potential with no significant effect on LOR entrapment efficiency percent in bilosomes. The elevation of particle size may be attributed to iron oxide nanoparticle incorporation, whereas the reduction in the absolute value of zeta potential is most likely caused by the positively charged iron oxide nanoparticles sticking to the bilosomes’ surface, which may have partially reduced the vesicles’ negative charge [75].

#### Magnetic properties

**Fig. 4:**
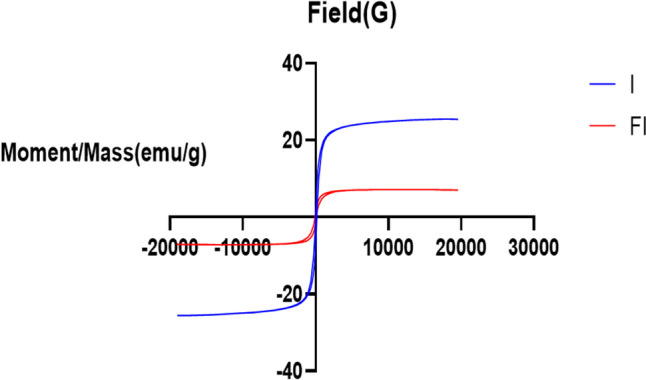
Hysteresis loop of the prepared (I; SPIONs) and (FI; LSB). Abbreviations: LSB, LOR SPION-loaded bilosome; SPION; superparamagnetic iron oxide nanoparticle; and LOR, lornoxicam

The saturation magnetization of SPIONs and LSB was 25.539 emu/g and 7.1805 emu/g, respectively, according to the plots of hysteresis loops (Fig. 4). All of the samples’ magnetic hysteresis curves intersected the origin, indicating that coercivity and remanent magnetization were both zero. This outcome demonstrated that both prepared SPIONs and LSB have superparamagnetic nature. Even though LSB’s saturation magnetization was smaller than SPIONs’, it still demonstrated significant magnetic responsiveness to outside magnetic fields. The causes of this are the entrapment of SPIONs within the bilosomes [76, 77].

The fact that these entrapped nanoparticles can still be influenced by an external magnetic field makes it possible to target specific sites by applying an external magnetic field [78].

### Morphological screening

**Fig. 5:**
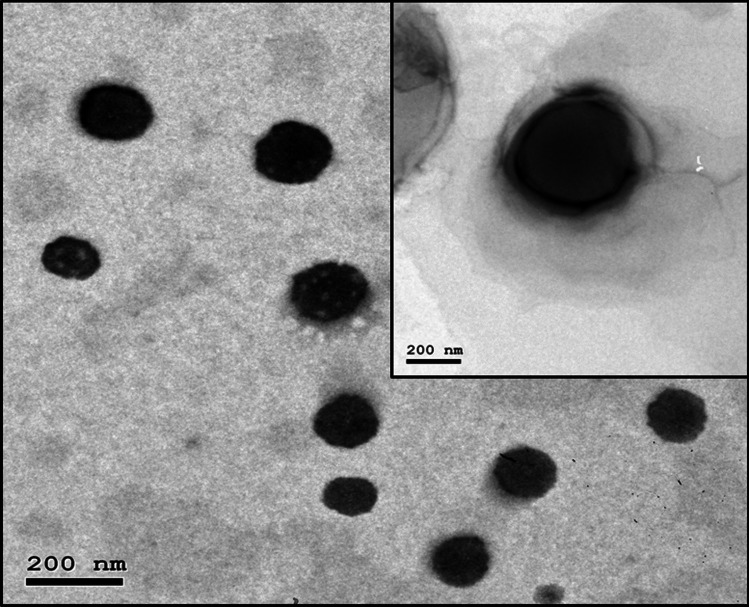
TEM photomicrograph of selected the vesicle (LB4). The inset shows the TEM image of (LSB). Abbreviations: LB4, the optimized LOR-loaded bilosome; LSB, LOR SPION-loaded bilosome; and LOR, lornoxicam

The TEM micrographs of the optimized LOR-loaded bilosome (LB4) and LSB are demonstrated in Fig. 5. The formulated bilosomes were spherical, unilamellar, uniform vesicles with no clustering. Moreover, the mean diameter of vesicles recorded by TEM was in agreement with the one obtained by the Zetasizer.

The LSB nanoparticles demonstrated a capsule-like structure with peripheral localization to indicate that the SPIONs were successfully integrated into the bilosomes [79].

### Differential scanning calorimetry

**Fig. 6:**
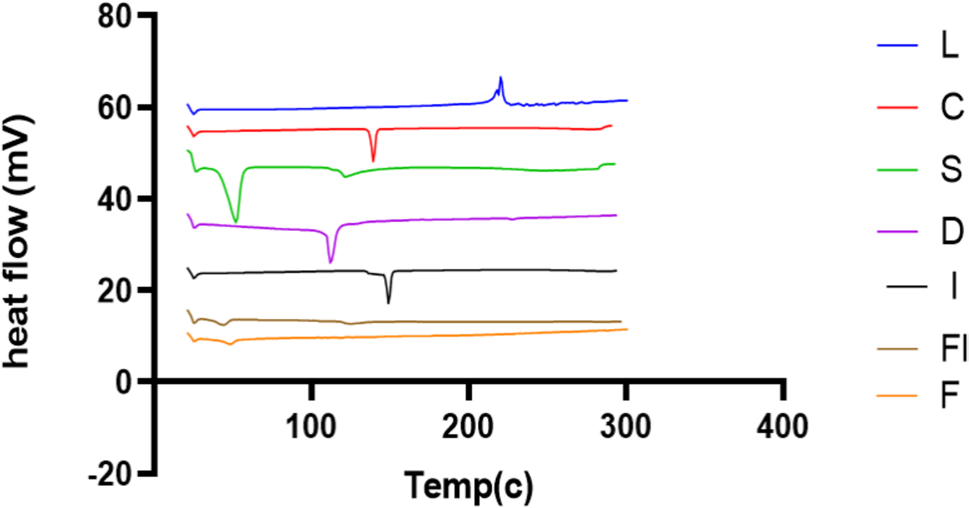
DSC thermograms of lornoxicam, cholesterol, Span 60, sodium deoxycholate, the optimized LOR-loaded bilosome (LB4), and LOR SPION-loaded bilosome (LSB)

Figure 6 shows the thermograms of the pure LOR, Span^®^ 60, cholesterol, SPION, the optimized lyophilized LOR-loaded bilosome (LB4), and lyophilized LSB. The DSC scan of the pure LOR showed a sharp strong exothermic peak at 220.32 °C, which is in the range of lornoxicam’s melting point (220–230 °C), and the sharp exothermic peak confirms the drug’s crystallinity [80]. According to DSC findings, cholesterol, Span 60, SDC, and SPIONs had crystal structures with sharp endothermic peaks at 148.09, 59.31, 118.45, and 157.5 °C, respectively, corresponding to the melting points of each material [81–84]. The DSC of Span 60 showed two distinct peaks: the first one at 59.32 °C representing the melting point in its crystal structure and the second one at 129.62 °C which displayed the flash point.

The DSC thermogram of the bilosomal system (optimized LOR-loaded bilosome (LB4) and LSB) revealed the disappearance of the peaks for all system’s components, which indicates the entrapment of each of the drug and SPION within bilosomes and their change to an amorphous form. The absence of the drug’s exothermic peak might be attributed to the interaction with the vesicle’s surfactant bilayers, according to an earlier study [72]. The SDC peak’s disappearance indicates that it has been fluidized in the surfactant’s lipid bilayer and that vesicles have developed. Peaks of both Span^®^ 60 and CHL vanished, indicating that CHL, SDC, and surfactant interact (drug solubilization in the dispersion of bilosome and its transition in an amorphous state) [72].

### Stability study

During storage, the optimized LOR-loaded bilosome (LB4) and LSB were observed to maintain their appearance with no signs of phase separation or aggregation. Furthermore, there was a nonsignificant reduction in the values of entrapment efficiency percent and zeta potential for both systems, and a nonsignificant rise in the values of particle size for the optimized LOR-loaded bilosome (LB4), and a nonsignificant reduction in the values of particle size of LSB which may be attributed to the modification of mean intensity distribution of the size (the particle’s intensity weighted distribution is used to compute the *z*-average) [85]. The responses of both stored and freshly prepared systems showed no significant changes (*p* > 0.05) and are presented in Table 3. These results verified that both systems were stable under the predetermined conditions of storage with no notable alterations.

**Table 3:** Effect of storage on the physical properties of LB4 and LSB

**Parameter**	**LB4 fresh**	**LB4 after 90 days at 4 ℃**	**LSB fresh**	**LSB after 90 days at 4 ℃**
**EE%**	87.10 ± 1.67	84.12 ± 0.12	87.24 ± 0.85	86.83 ± 0.15
**PS (nm)**	254 ± 14	259 ± 16	323 ± 7.0	317 ± 3.0
**ZP (mV)**	-40.23 ± 0.13	-36.87 ± 0.21	-32.47 ± 1.60	-30.63 ± 0.13

Data are presented as mean ± SD (*n* = 3)

*EE%* entrapment efficiency percent, *PS* particle size, *ZP* zeta potential, *LB4* the optimized LOR-loaded bilosome, *LSB* selected bilosome loaded with SPION

### Preparation of LSB in situ forming hydrogels

In the current study, the cold method rather than the hot method was conducted for the preparation of in situ forming hydrogel because of the better solubility of the polymer in the cold system due to the formation of hydrogen linkage at low temperature which results in the formation of a clear solution [86, 87].

Synperonic™ PE/F 127 was chosen based on its thermogelling behavior as it exists as a liquid at low temperatures but transforms into a gel at high temperatures [26]. Different concentrations of PE/F 127 were evaluated (Table 4) to find the formulation that retains its solution nature at room temperature to be readily injected and turns into a gel after injection in the body. As presented in Table 4, formulations with PE/F 127 concentration ranging from 16 to 25% had the ability to form a gel but formula with 15% PE/F 127 could not form a gel. This finding might be because PE/F 127 has a relatively limited concentration range that is appropriate for application, around 16–20% (w/v). The preparation cannot form a gel and retain its liquid state regardless of the temperature (both physiological (37 °C) and non-physiological (4 °C)) when the concentration is less than 16% (w/v). When exceeding 20% (w/v), it was converted into a gel at ambient temperature and exhibited no free-flowing features at 4 °C, resulting in inconvenient usage and storage [88, 89].

**Table 4:** Composition and gelation characteristics of in situ hydrogel formulations

**Formulations**	**PE/F127** **(%w/v)**	**HA** **(%w/v)**	**Gelation**	**Gelation temperature** **(Tsol/gel) (°C)**
**LSB1**	25	0.20	yes	22 ± 0.40
**LSB2**	18	0.20	yes	28 ± 0.22
**LSB3**	17	0.20	yes	32 ± 0.37
**LSB4a**	16	0.20	yes	34 ± 0.45
**LSB5**	15	0.20	no	-
**LSB4b**	16	0.15	yes	36 ± 0.36
**LSB4c**	16	0.10	yes	37 ± 0.29

Data are presented as mean ± SD (*n* = 3)

*LSB *LOR/SPION-loaded bilosome, *PE/F 127* Synperonic™ PE/F 127, *HA* hyaluronic acid

### Characterization of LSB in situ forming hydrogels

#### Selection of the optimized in situ forming hydrogel based on the gelation temperature

An ideal thermosensitive system should have a sol-gel transition temperature above ambient temperature, ideally 30 °C, and will gel upon injection at (37 °C) [89, 90]. As presented in Table 4, LSB1 and LSB2 showed a sol-gel temperature of 22 ± 0.4 and 28 ± 0.22 °C, respectively, and therefore are not suitable for in situ forming hydro gelling utilization. On the other side, other systems exhibited acceptable gelation temperatures in the range of 32–39 °C. It was noticed that the increase in the concentration of PE/F 127 from 16% w/v (LSB4) to 25% w/v (LSB1) was accompanied by a decrease in the gelation temperature. PE/F 127 is an amphiphilic invertible thermogelling triblock copolymer composed of a lipophilic polypropylene oxide (PPO) monomeric unit sandwiched between two hydrophilic polyethylene oxide (PEO) monomeric units; both participate in the micellar agglomeration phenomenon [91]. Amphiphilic triblock copolymer molecules can form tiny micellar subunits in aqueous liquids. Above a certain concentration, critical micelle concentration that is known as the concentration of monomers at which micelles developed, and temperature, critical micellization temperature (CMT) that is known as the temperature below which amphiphile is present as unimer and unimers and lumps coexist above that, the polymer molecules assemble to form a large micellar crosslinked network [92]. Below CMT, both propylene and ethylene oxide units are hydrated, and propylene oxide is slightly soluble in aqueous solutions. Moreover, exceeding CMT, the PPO blocks turn into dehydrated form and therefore less soluble than PEO blocks, which causes hydrophobic interactions between the PPOs and the development of spherical micelles with an inner dehydrated PPO core and an external tumid hydrous PEO shell [43]. Such micelles are challenging to disperse individually in the solution; rather, they interact with one another and entangle to create a three-dimensional network structure [93].

Different concentrations of HA solution were applied to the thermosensitive in situ forming hydrogelling system to examine HA’s impact on the PE/F 127 system. It is worthy to note that the sol-gel transition temperature has been shifted to higher temperatures (from 34 to 37 °C) with decreasing the concentration of HA from 0.2 to 0.1 w/v% [94]. This is probably because the addition of high-molecular-weight HA could improve the dense packing of pluronic micelles at temperature of gelation above the lower critical solution temperature through lipophilic interaction among the CH3CO-branch on HA and the CH3-branch on PE/F127 resulting in the crosslinking among micelles and the reduction of the critical gelation temperature value of the thermosensitive hydrogel [95]. Generally, the temperature recommended for the creation of an in situ forming hydrogel system for injectable application is 37 °C, so the LSB in situ forming hydrogel (16 w/v% Synperonic™ PE/F 127 and 0.1 w/v% HA) with a gelation temperature of 37 ± 0.29 °C was selected as an ideal in situ forming hydrogel for the delivery of LOR.

#### Evaluation of the optimized in situ forming hydrogel formula

##### Assessment of gelation time

In the current study, it was observed that the optimized LSB in situ forming hydrogel (LSB4c) recorded a short gelation time of about 38 ± 1.6 s, which is suitable for injection.

##### Assessment of viscosity and rheological properties

At 10 rpm, the viscosity of the optimized LSB in situ forming hydrogel (LSB4c) at 4 °C and 37 °C was 79.5 ± 7.33 cps and 325.3 ± 11.03 cps, respectively. The previous results indicate that the viscosity increased about four times when the temperature was shifted from 4 to 37 °C due to gelation.

**Fig. 7:**
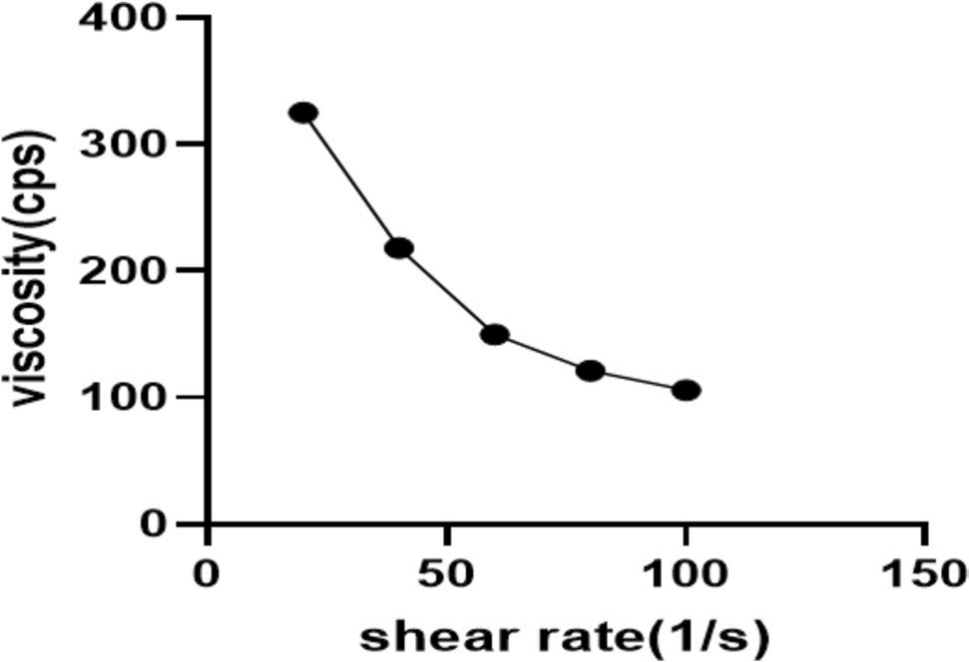
Rheological characterization of LSB4c (a plot of viscosity and shear rate). The measurements were performed at 37 ± 2 °C, at varying shear speeds (10–50 rpm) with 10 s between every two consecutive speeds. Abbreviations: LSB4c, the optimized LOR SPION-loaded bilosome in situ hydrogel system

According to the rheological analysis of the formula, the optimized LSB in situ forming hydrogel (LSB4c) displayed shear-thinning pseudoplastic flow, as illustrated in Fig. 7. It is worthy to note that the pseudoplastic flow of hydrogel is ideal for injectable preparations since a high shear rate during injection will reduce the hydrogel’s viscosity to make it easier to inject, while the low shear rate will cause the hydrogel to keep its regular structure [96]. This flow pattern might be explained by the entanglement of the polymeric molecules that comprise the optimized formula during relaxation. Upon exposure to shear stress, the molecules detangle and align themselves with the flow direction resulting in the reduction of flow resistance along with the release of part of the trapped solvent. That accounts for the decreased viscosity [96, 97].

##### Syringeability study

The optimized LSB in situ forming hydrogel (LSB4c) was found to be syringeable as it was smoothly injected through the 21-G needle at a cold temperature, assuring the convenience of administration during usage.

### In vitro release study

**Fig. 8:**
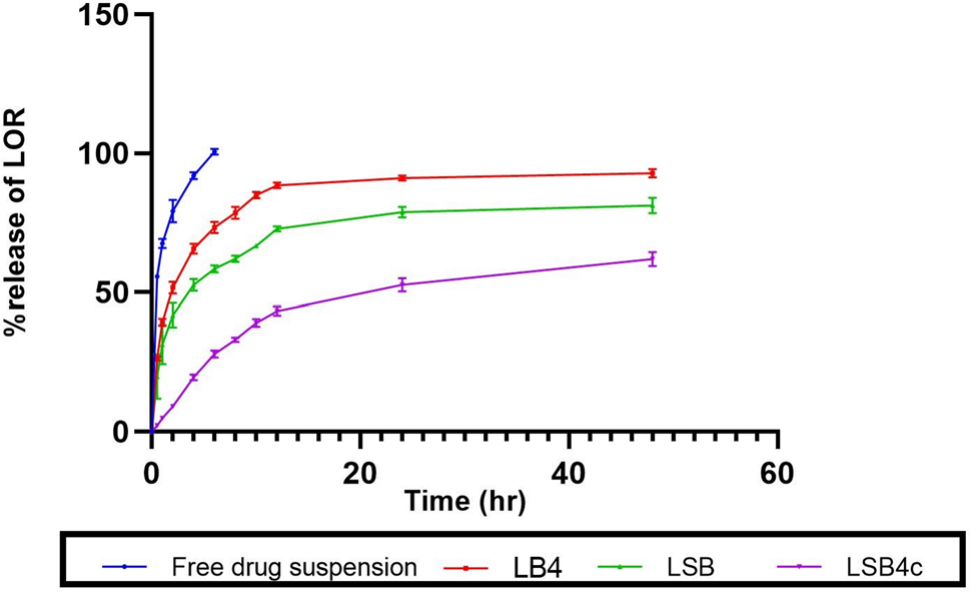
LOR release from LB4, LSB, LSB4c, and the free drug suspension. Abbreviations: LOR, lornoxicam; LB4, the optimized LOR-loaded bilosome, LSB, LOR SPION-loaded bilosome; and LSB4c, the optimized LOR SPION-loaded bilosome in situ hydrogel system

Figure 8 demonstrates the LOR release from the optimized LOR-loaded bilosome (LB4), LSB, the optimized LSB in situ forming hydrogel (LSB4c), and the free drug suspension. From the figure, it could be noticed that the free drug suspension revealed a 100% drug release within 6 h while all other formulations showed an obvious sustainment in drug release with the demonstration of a biphasic release pattern. First, there was a burst release in the first 6 h, which may be attributed to the free drug in the dispersion medium and the loosely attached drug molecules to the bilosomal surface [86]. The first phase was followed by a steady release where 92.87 ± 1.51%, 81.14 ± 2.82%, and 61.97 ± 2.49% of LOR were liberated from the optimized LOR-loaded bilosome (LB4), LSB, and the optimized LSB in situ forming hydrogel (LSB4c) after 48 h, respectively. The ability of bilosomes to function as drug reservoirs and release the encapsulating drugs in a sustained and controlled way is thought to be the cause of the observed sustained release pattern from the bilosomal dispersions [87]. The slower release of the drug from LSB in contrast to the optimized LOR-loaded bilosome (LB4) (*p* = 0.031) is likely due to the increased particle size of the former which in turn decreased the exposed surface area, thereby decelerating the drug release rate [29].

Loading LSB into an in situ forming hydrogel system resulted in prolonged release of LOR compared with LSB alone (*p* = 0.0001). This is because the in situ forming hydrogel system offers an additional obstacle that greatly hinders the diffusion of LOR to the external dissolution medium [98].

The analysis of LOR release kinetics from the developed nanoparticles was done using various kinetic models, namely first order, zero order, Hixson-Crowell, Higuchi, and Korsmeyer-Peppas. The Korsmeyer-Peppas model was chosen as the best kinetic release model because it showed the highest *R*^2^ value [99]. In the Korsmeyer-Peppas model, the exponent of release *n* was estimated, indicating the release mechanism of the drug. If *n* is equal to 0.45, then the release of the drug complies case I or Fickian diffusion mechanism, *n* greater than 0.45 but below 0.89 indicates anomalous behavior or non-Fickian diffusion, *n* equal to 0.89 indicates case II transport, and *n* greater than 0.89 indicates super case II transport [48, 100]. The values of *n* for all generated formulations were observed to be ≤ 0.45, pointing out that the drug release mechanism is Fickian diffusion (unpublished data).

### In vivo studies

In this study, the IM injection of the free LOR in situ forming hydrogel, the optimized LOR-loaded bilosome (LB4) in situ forming hydrogel, or the optimized LSB in situ forming hydrogels (LSB4c) was tested in rats with carrageenan-induced joint inflammation. Regarding the group treated with the optimized LSB in situ forming hydrogel (LSB4c), an external magnet was directed toward the knee to attract the nanoparticles to its site of action with the aim of improving its effectiveness.

#### Effect on joint diameter

**Fig. 9:**
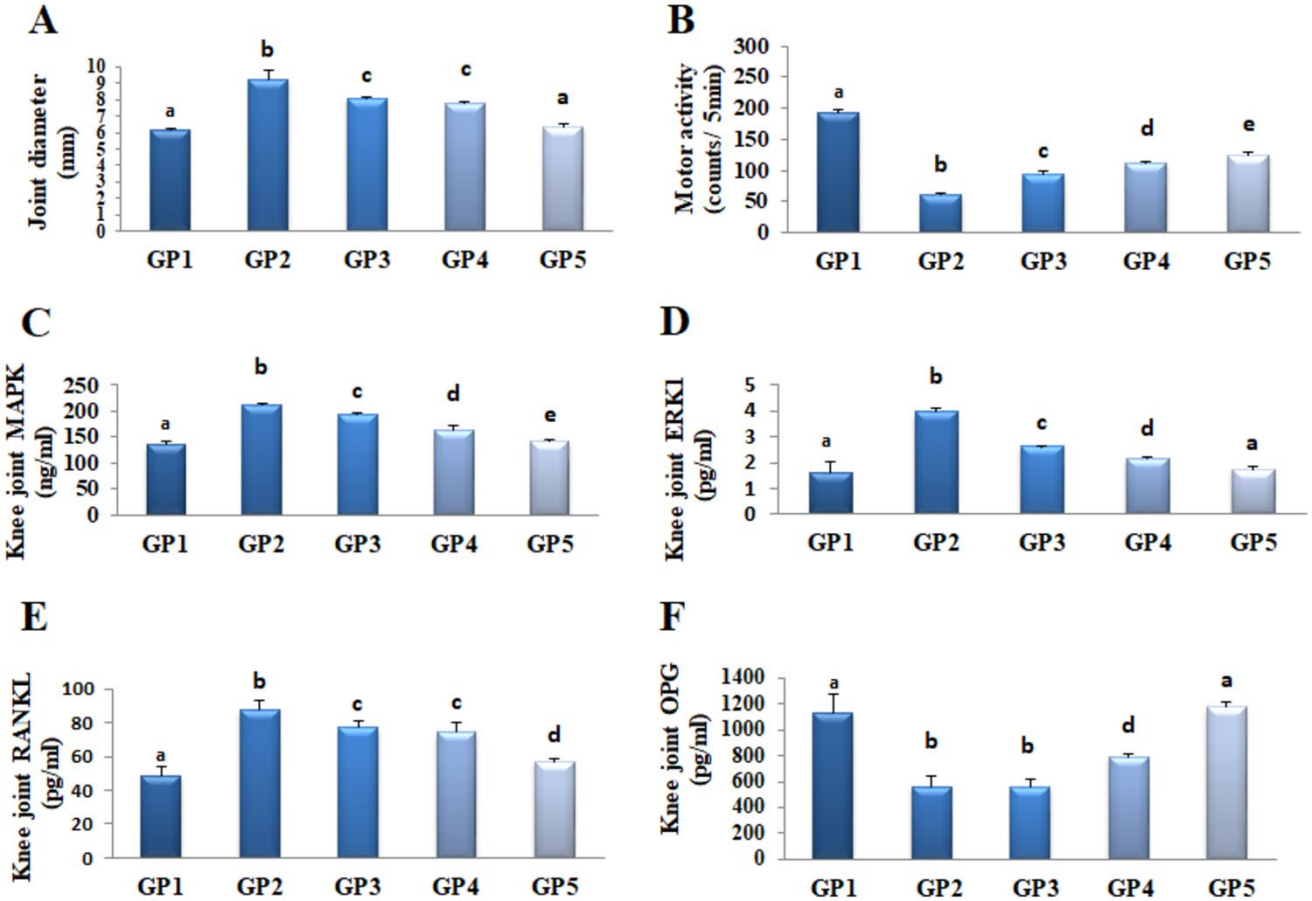
**A** Effect on joint diameter, **B** locomotor activity, **C** MAPK, **D** ERK1, **E** RANKL, and **F** OPG. Data were expressed as mean ± SD (*n* = 8). Statistical analysis was carried out by one-way ANOVA followed by Fisher’s LSD test for multiple comparisons. Different letters are significantly different at *p* < 0.05

Results revealed that the diameter of the right knee joints was significantly elevated by 52% in osteoarthritic rats compared to the normal control. Treatment of the rat knee joint by IM injection of the free LOR in situ forming hydrogel (GP3), the optimized LOR-loaded bilosome (LB4) in situ forming hydrogel (GP4), or the optimized LSB in situ forming hydrogel (LSB4c) (GP5) succeeded in improving the inflammation caused by carrageenan. Results confirmed that the right knee joint diameter in rats that received treatment with the free LOR, the optimized LOR-loaded bilosome (LB4), and the optimized LSB in situ forming hydrogel (LSB4c) decreased by 12%, 16%, and 31%, respectively, as compared to the positive control group that received carrageenan with no treatment (GP2). In addition, treatment with the optimized LSB in situ forming hydrogel (LSB4c) reduced the rat joint diameter by 22% and 18% as compared to free drug and the optimized LOR-loaded bilosome (LB4), respectively, and returned it to its normal value (Fig. 9A; Table 5).

**Table 5:** Effect of different formulas on joint diameter, locomotor activity, and various biomarkers

	**GP1**	**GP2**	**GP3**	**GP4**	**GP5**
**Joint diameter (mm)**	6.11 ± 0.11	9.26 ± 0.54	8.11 ± 0.05	7.76 ± 0.14	6.36 ± 0.15
**Locomotor activity (counts/5 min)**	193.0 ± 5.0	59.6 ± 3.0	92.8 ± 6.0	112.6 ± 2.0	125.0 ± 3.0
**MAPK (ng/mL)**	135 ± 5	213 ± 3	193 ± 3	164 ± 9	143 ± 2
**ERK1 (pg/mL)**	1.61 ± 0.40	3.96 ± 0.12	2.62 ± 0.01	2.17 ± 0.07	1.75 ± 0.11
**RANKL (pg/mL)**	48.81 ± 5.13	87.18 ± 5.95	76.80 ± 4.55	74.25 ± 6.15	56.92 ± 1.59
**OPG (pg/mL)**	1134 ± 139	561 ± 84	552 ± 71	794 ± 23	1176 ± 46

**% Reduction/improvement or elevation**					
** Joint diameter (mm)**	−	+ 52	− 12	− 16	− 31
**Locomotor activity (counts/5 min)**	−	− 69	+ 56	+ 89	+ 110
** MAPK (ng/mL)**	−	+ 58	− 9	− 23	− 33
** ERK1 (pg/mL)**	−	+ 146	− 34	− 45	− 56
** RANKL (pg/mL)**	−	+ 79	− 12	− 15	− 35
** OPG (pg/mL)**	−	− 51	+ 2	+ 42	+ 110

Data are presented as mean ± SD (*n* = 3)

*GP1 *normal control, *carrageenan group, Gp2* positive control, *Gp3* rats injected IM with in situ hydrogel of free drug, *GP4* rats injected IM with in situ hydrogel of the optimized LOR-loaded bilosome (LB4), *GP5* the optimized LSB in situ forming hydrogel (LSB4c), *MAPK* mitogen-activated protein kinase, *ERK1* extracellular signal-regulated kinase, *RANKL* receptor activator of nuclear factor kappa beta, *OPG* osteoprotegerin. Positive sign (+) indicates improvement while a negative sign (−) indicates a reduction upon comparing GP2 versus GP1 and GP3, GP4, and GP5 versus GP2

#### Effect on locomotor activity

Osteoarthritis arises from an imbalance between the chondrocyte synthesis and degeneration processes inducing loss in cartilaginous tissue, normal joint function, and locomotor activity [101]. Arthritis induced by carrageenan reduced locomotor activity by 69%, as compared to normal values (negative control). Treatment with free drug, the optimized LOR-loaded bilosome (LB4), and the optimized LSB in situ forming hydrogel (LSB4c) elevated locomotor activity by 56%, 89%, and 110%, respectively, as compared to the carrageenan group. In addition, treatment with the optimized LSB in situ forming hydrogel (LSB4c) elevated locomotor activity by 35% and 11% as compared to free drug and the optimized LOR-loaded bilosome (LB4), respectively (Fig. 9B; Table 5).

#### Effect on MAPK/ERK1 signaling pathway

Carrageenan induced inflammation and arthritis in rats through MAPKs/ERK signaling pathway [102]. MAPKs and ERK inflammatory kinases provoke inflammatory and neuropathic pain in the dorsal root ganglion and spinal cord. In addition, ERK enhances neutrophil infiltration into the synovium in arthritis [103, 104]. In the current study, carrageenan injection induced an elevation in MAPK/ERK1 signaling pathway by 58% and 146%, respectively, as compared to normal values. Treatment with free drug, the optimized LOR-loaded bilosome (LB4), and the optimized LSB in situ forming hydrogel (LSB4c) reduced MAPK joint content by 9%, 23%, and 33% and ERK1 content by 34%, 45%, and 56%, respectively, as compared to the carrageenan group. In addition, treatment with the optimized LSB in situ forming hydrogel (LSB4c) reduced MAPK and ERK1 levels by 26% and 33%, respectively, compared to the free drug, and by 13% and 20%, respectively, compared to the optimized LOR-loaded bilosome (LB4) suggesting that the optimized LSB in situ forming hydrogel (LSB4c) has a superior suppressor effect on MAPK/ERK1 signaling pathway as it returned ERK1 to its normal values (Fig. 9C, D; Table 5). These results are in line with a previous study that showed that lornoxicam-loaded bilosomes using 3^1^.2^2^ full factorial design modulate MAPK/ERK1 for the management of osteoarthritis [32].

#### Effect on RANKL/OPG in rats’ knee joints

RANKL is the osteoclastogenic cytokine leading to bone resorption, while OPG inhibits bone resorption via its suppressor effect on RANKL [7]. A significant elevation of RANKL concentration in knee joints was noticed in rats injected intra-articularly with carrageenan by 79% versus the normal control group. The free drug, the optimized LOR-loaded bilosome (LB4), and the optimized LSB in situ forming hydrogel (LSB4c) treatments ameliorated this elevation by 12%, 15%, and 35% respectively versus the carrageenan control group. In addition, treatment with the optimized LSB in situ forming hydrogel (LSB4c) reduced RANKL levels by 26% and 23% respectively compared to the free drug and the optimized LOR-loaded bilosome (LB4). Conversely, a significant reduction by 51% of OPG concentration in knee joints was noticed in rats injected with carrageenan versus the normal control group. The optimized LOR-loaded bilosome (LB4) and the optimized LSB in situ forming hydrogel (LSB4c) treatments modulated this reduction by 42% and 110% respectively versus the carrageenan control group. Moreover, the treatment with the optimized LSB in situ forming hydrogel (LSB4c) increased OPG levels by 113% and 48% compared to free drug and the optimized LOR-loaded bilosome (LB4) respectively and returned its value to normal level (Fig. 9E, F; Table 5). Our results revealed that the optimized LSB in situ forming hydrogel (LSB4c) has an anti-arthritic effect for the first time via controlling RANKL/OPG joint content.

#### Histopathological evaluation

**Fig. 10:**
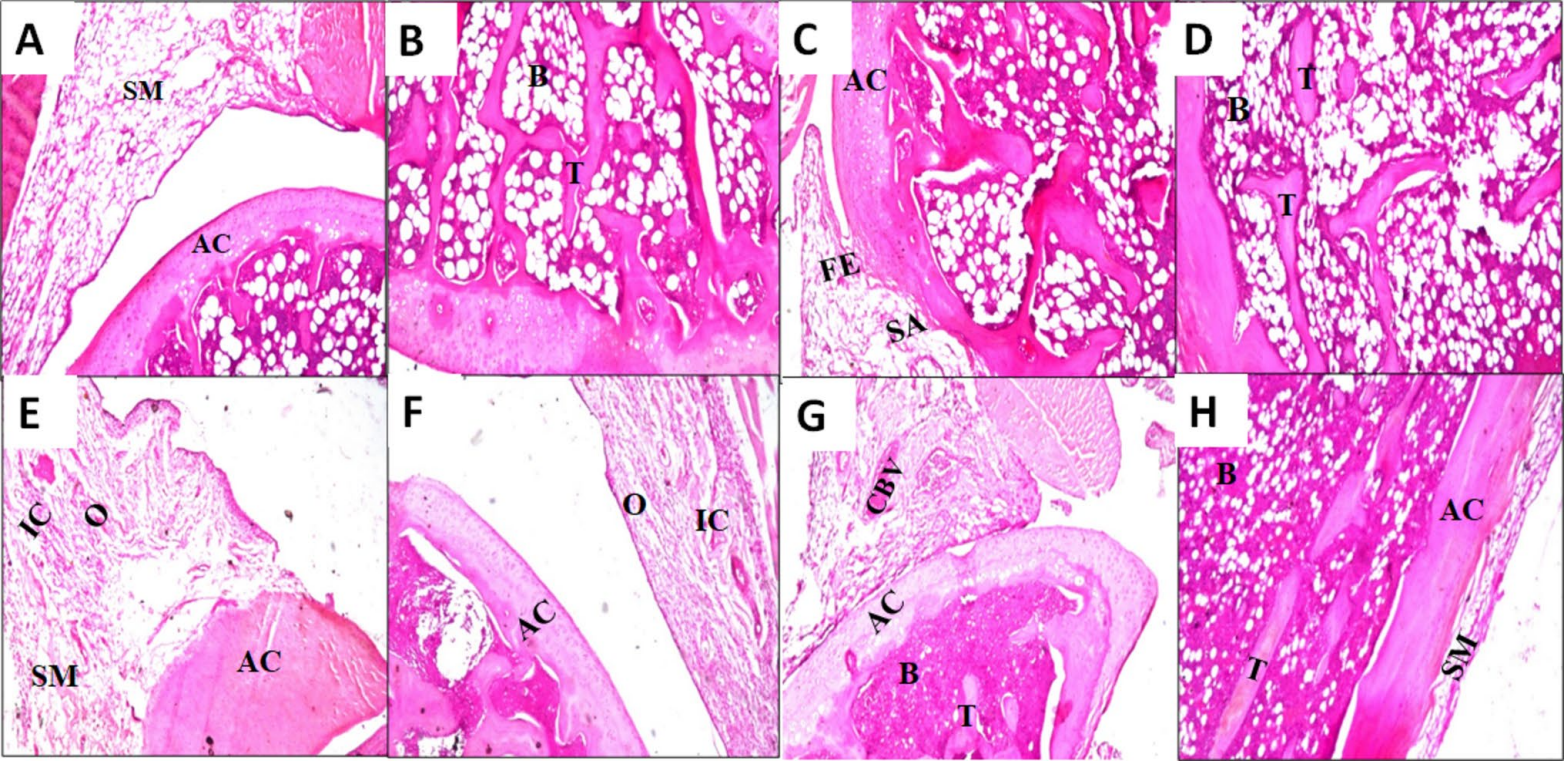
Photomicrographs of **H** & **E**–stained histological sections of normal, carrageenan, free LOR, the optimized LOR-loaded bilosome (LB4), and the optimized LOR SPION-loaded bilosomes in situ forming hydrogel (LSB4c) treated knee joints: **A**, **B** normal control; **C**, **D** positive control (carrageenan group); **E**, **F** free LOR; **G** the optimized LOR-loaded bilosomes (LB4); and **H** the optimized LOR SPION-loaded bilosomes in situ forming hydrogel (LSB4c). Abbreviations: B, bone marrow; T, trabeculae; SM, synovial membrane, AC, articular cartilage; O, edema; IC, inflammatory cell; FE, focal erosion; SA, synovial adhesion; CBV, congested blood vessel

The histopathological characterization of knee joints of rats from various groups is demonstrated in Fig. 10. The normal histopathological features are observed for the negative control group regarding the synovial membrane, articular cartilaginous surface, bone marrow (Fig. 10A), or bone trabeculae (Fig. 10B), while the group of experimentally positive control rats revealed that focal erosion was detected in the articular cartilaginous surface associated with synovial membrane adhesion (Fig. 10C). In addition, the bone trabeculae showed osteoporosis (Fig. 10D).

Administering the free drug did not provide any relief of the inflammation caused by carrageenan. As demonstrated in (Fig. 10E), edema with inflammatory cell infiltration was detected in the synovial membrane. However, the articular cartilaginous surface showed normal histological structure (Fig. 10F). In the group of rats that underwent experimental induction and were treated with the optimized LOR-loaded bilosome (LB4), histopathology revealed the presence of congestion in the blood vessels of the synovial membrane, but the articular cartilage appeared normal (Fig. 10G). On the other hand, the group of rats that were experimentally induced and treated with the optimized LSB in situ forming hydrogel (LSB4c) showed that the synovial membrane and articular cartilaginous surface had a normal histological structure, as seen in Fig. 10H. These findings indicate that the application of an external magnet to the knee after intra-muscular administration of LSB into the thigh muscle facilitates targeting and localization of LOR/SPION-loaded nanoparticles and maximizes their concentration in the knee joint resulting in the optimum management of OA.

## Conclusion

LOR-loaded bilosomes were successfully fabricated via the thin film hydration method and optimized by varying the surfactant type besides the type and amount of bile salt. All the developed bilosomes were within the nanosized range with high LOR encapsulation. The optimized LOR-loaded bilosome (LB4) displayed the highest %EE of the drug with a favorable size and zeta potential; thus, it was chosen to be loaded with SPIONs (LSB). The prepared LSB system was loaded into an injectable in situ forming hydrogel containing Synperonic™ PE/F 127 (PE/F127) and hyaluronic acid (HA). The in vivo results showed that the optimized LSB in situ forming hydrogel (LSB4c) has superiority over the optimized LOR-loaded bilosome (LB4) and the free drug hydrogel in the management of inflammation and osteoarthritis with a significant elevation in OPG level and reduction in RANKL, MAPK, and ERK1 levels as well as significant enhancement in the histopathological evaluation of the knee joint. Hence, the LOR SPION-loaded bilosome in situ forming hydrogel system that is injected intra-muscularly into the thigh muscle combined with the application of an external magnet to the knees could be regarded as a competent platform for the suppression of OA in addition to being a safe and more acceptable alternative to intra-articular injection.

## Data Availability

The datasets generated and/or analyzed during the current study are available from the corresponding author on reasonable request.
